# Participation of *Trypanosoma cruzi* gp63 molecules on the interaction with *Rhodnius prolixus*

**DOI:** 10.1017/S0031182019000441

**Published:** 2019-05-06

**Authors:** Karina M. Rebello, Livia A. Uehara, Vítor Ennes-Vidal, Aline S. Garcia-Gomes, Constança Britto, Patrícia Azambuja, Rubem F. S. Menna-Barreto, André L. S. Santos, Marta H. Branquinha, Claudia M. d'Avila-Levy

**Affiliations:** 1 Laboratório de Estudos Integrados em Protozoologia, Instituto Oswaldo Cruz (IOC), Fundação Oswaldo Cruz (FIOCRUZ), Rio de Janeiro, Brazil; 2 Instituto Federal de Educação, Ciência e Tecnologia do Rio de Janeiro (IFRJ), Rio de Janeiro, Brazil; 3 Laboratório de Biologia Molecular e Doenças Endêmicas, Instituto Oswaldo Cruz (IOC), Fundação Oswaldo Cruz (FIOCRUZ), Rio de Janeiro, RJ, Brazil; 4 Laboratório de Bioquímica e Fisiologia de Insetos, Instituto Oswaldo Cruz (IOC), Fundação Oswaldo Cruz (FIOCRUZ), Rio de Janeiro, Brazil; 5 Laboratório de Biologia Celular, Instituto Oswaldo Cruz, Fundação Oswaldo Cruz, Rio de Janeiro, Brazil; 6 Laboratório de Estudos Avançados de Microrganismos Emergentes e Resistência, Instituto de Microbiologia Paulo de Góes, Universidade Federal do Rio de Janeiro, Rio de Janeiro, Brazil; 7 de Duve Institute, Université Catholique de Louvain, Brussels, Belgium

**Keywords:** Insect, leishmanolysin, invertebrate host, major surface peptidase (MSP), triatomine, vector

## Abstract

*Trypanosoma cruzi* is the causative agent of Chagas disease, a vector-borne disease. The parasite molecules involved in vector interaction have been little investigated. Metallopeptidases and gp63 molecules have been implicated in parasite adhesion of several trypanosomatids to the insect midgut. Although gp63 homologues are highly expanded in the *T. cruzi* genome, and are implicated in parasite–mammalian host interaction, its role in the insect vector has never been explored. Here, we showed that divalent metal chelators or anti-Tcgp63-I antibodies impaired *T. cruzi* adhesion to *Rhodnius prolixus* midgut. Parasites isolated after insect colonization presented a drastic enhancement in the expression of Tcgp63-I. These data highlight, for the first time, that Tcgp63-I and Zn-dependent enzymes contribute to the interaction of *T. cruzi* with the insect vector.

## Introduction

*Trypanosoma cruzi* is the aetiological agent of Chagas disease, a vector-borne disease transmitted by the bite of a triatomine bug, which affects 8 million people worldwide (Perez-Molina and Molina, 2018). In Brazil, vector transmission was drastically reduced by governmental policies of insect control, which were directed to *Triatoma infestans* (Abad-Franch *et al*., 2013). Nevertheless, other triatomine species are known to transmit *T. cruzi* (Noireau *et al*., 2009; Bern *et al*., 2011; Waleckx *et al*., 2015), and several others are on the merge of becoming competent vectors, to sum up, vector domiciliation is also an emerging threat (Salazar *et al*., 2015). The comprehension of the molecular mechanism of parasite–insect vector interaction can help to envision vector transmission control strategies.

Few studies have been performed on the characterization of *T. cruzi* molecules involved in the parasite adhesion, interaction and colonization of the insect. After a triatomine blood meal, replicative non-infective epimastigotes adhere and proliferate in the midgut, and subsequently migrate to the posterior midgut, where metacyclogenesis occurs, then non-proliferative metacyclic trypomastigotes can infect a mammalian host (Azambuja *et al*., 2005). Glycoinositolphospholipids (GIPLs) are the major molecules expressed on the epimastigote cell surface. These molecules are involved in the parasite adhesion to the luminal surface of the insect midgut and may be one of the determinants of the infection in the vector (Nogueira *et al*., 2007). In addition, Gp72, cruzipain and calpain-like molecules may also be involved in this part of the parasite life cycle (de Jesus *et al*., 1993; Basombrio *et al*., 2002; Ennes-Vidal *et al*., 2011; Uehara *et al*., 2012).

Peptidases, a class of hydrolytic enzymes responsible for breaking peptide bonds, has attracted the attention of distinct research groups because of their role in several crucial steps of the life cycle of the trypanosomatid parasites (Alvarez *et al*., 2012; Santos *et al*., 2012; Branquinha *et al*., 2013, *2015*; d'Avila-Levy *et al*., 2014). The most abundant and extensively studied metallopeptidase in trypanosomatids is gp63, which was first identified and described as an immunodominant protein from several *Leishmania* spp. (d'Avila-Levy *et al*., 2014), and it is a well-characterized virulence factor in *Leishmania* spp. (Olivier *et al*., 2012), which also takes a role in insect adhesion (Soares *et al*., 2017). Due to the strong recognition of gp63 and cruzipain by the sera from infected patients in the early 80s, the role of these molecules has been extensively investigated in leishmaniasis and Chagas disease, respectively. The biochemical characterization revealed their hydrolytic activity, which guided the peptidase research in each disease-causing parasite. For instance, in *T. cruzi* from the total of indexed articles in the Pubmed (http://www.ncbi.nlm.nih.gov/pubmed/) that are retrieved by the search string ‘peptidase’ and its synonyms, 50% accounts for articles related to cruzipain and its synonyms. In *Leishmania* spp., 37% of the scientific literature linked to peptidase accounts for gp63. Besides the historical discovery of each molecule, a routine zymography, which is the most popular technique for peptidase screening, reveals an abundant metallopeptidase in *Leishmania* spp. cellular extracts against an abundant cysteine peptidase in *T. cruzi* cellular extracts (Chaudhuri and Chang, 1988; Cazzulo *et al*., 1990). In this sense, it is not clear whether gp63 homologues in *T. cruzi* have a secondary role for parasite virulence or if cruzipain leaded research to the detriment of gp63. Genes encoding gp63 have been extensively amplified in the *T. cruzi* genome, and an indirect correlation between gene expansion and proteolytic activity in trypanosomatids is clearly found (d'Avila-Levy *et al*., 2014). Nevertheless, *T. cruzi* gp63 homologues involvement in mammalian host cells invasion have been demonstrated by two independent research groups (Cuevas *et al*., 2003; Kulkarni *et al*., 2009).

Here, we have assayed the role of *T. cruzi* metallo-dependent enzymes, particularly Tcgp63-I, on the *T. cruzi*–*R. prolixus* vector interaction. The influence of ion chelators on parasite viability, the capacity of adhesion to the vector midgut and expression of Tcgp63-I was analysed. Also, Tcgp63-I levels were evaluated after the protozoa colonization of *R. prolixus*. Collectively, the results indicate the participation of metallo-dependent enzymes and Tcgp63-I on the interaction with the insect vector and add new insights into the functional role of Tcgp63-I in *T. cruzi* epimastigotes.

## Materials and methods

### Abbreviations, chemicals and buffer composition

BHI – 3.7% brain heart infusion medium; BSA – bovine serum albumin; Cha – cyclohexylalanine; CHAPS – 3-((3-cholamidopropyl)dimethylammonio)-1-propanesulphonate; Dpa – *N*-3-(2, 4-dinitrophenyl)-L-2,3-diaminopropionyl; DMSO – dimethyl sulphoxide; DTT – dithiothreitol; E-64 – *trans*-epoxysuccinyl-L-leucylamido(4-guanidino)butane; EDTA – ethylenediaminetetraacetic acid; EGTA – ethylene glycol-bis(*β*-aminoethyl ether)-*N*,*N*,*N*′,*N*′-tetraacetic acid; FBS – heat-inactivated fetal bovine serum; MCA – (7-methoxycoumarin-4-yl)acetyl; Nva – norvaline; PBS – phosphate buffer saline, 150 mm NaCl, 20 mm phosphate buffer, pH 7.2; PMSF – phenylmethylsulphonyl fluoride; RSB – ‘Rhodnius saline buffer’ (160 mm KCl, 100 mm D-glucose, 4.2 mm NaHCO_3_, pH 7.8); SDS-PAGE – sodium dodecyl sulphate-polyacrylamide gel electrophoresis; SDS-PAGE sample buffer (62 mm Tris-HCl, pH 6.8, 2% SDS, 25% glycerol, 0.01% bromophenol blue and 1 mm *β*-mercaptoethanol); TBST – Tris buffer saline (150 mm NaCl; 10 mm Tris, pH 7.4 containing 0.05% Tween 20); TLCK – N*α*-tosyl- L-lysine chloromethyl ketone hydrochloride; White's solution-HgCl_2_ 0.25 g, NaCl 6.5 g, HCl 1.25 mL, 250 mL of ethanol, H_2_O qsp 1000 mL.

### Parasite strains and cultivation

The following *T. cruzi* isolates were obtained from the Coleção de Protozoários da Fundação Oswaldo Cruz (COLPROT-FIOCRUZ, http://colprot.fiocruz.br): Dm28c (COLPROT 010), G (COLPROT 216), Y (COLPROT 106) and CL Brener (COLPROT 005). The epimastigote forms were grown in BHI medium, containing 0.002% hemin, supplemented with 10% FBS, at 28 °C for 4 days, to reach late-log growth phase.

### Effect of divalent metal chelators on parasite growth and viability

To this set of experiments, 2 × 10^7^ *T. cruzi* Dm28c epimastigotes were collected from late-log growth cultures by centrifugation (1500 × ***g*** for 5 min at 4 °C), washed three times in PBS and incubated either in RSB for 1 h, or in BHI medium for 24–96 h in the presence of divalent metal chelators (EDTA, EGTA and phenanthroline) at concentrations ranging from 0.5 to 100 *µ*m. Control parasites were incubated with no compound. Parasites treated with the highest DMSO dose used to dissolve phenanthroline were assessed in parallel for the solvent control. Afterwards, the percentage of viable parasites was estimated by trypan blue dye exclusion and counting of actively motile parasites in a Neubauer chamber. Cell viability was expressed as percentage in relation to control cells, assigned as 100%. The 50% inhibitory concentration (IC_50_), i.e. the minimum drug concentration that caused a 50% reduction in survival/viability in comparison with that in identical cultures without the compound, was evaluated after 1 h of exposure. These values were determined by linear regression analysis by plotting the number of viable epimastigotes *vs* log drug concentration.

### Parasite treatment prior to insect interaction and flow cytometry analyses

*Trypanosoma cruzi* Dm28c parasites (2 × 10^7^ cells in 100 *µ*L RSB) were pretreated or not for 1 h with different modulators: (1) divalent metal chelators (phenanthroline, EDTA and EGTA) at concentrations ranging from 0.2 to 1 *µ*m; (2) anti-Tcgp63-I antibodies (kindly provided by Dr Daniel O. Sánchez, Universidad Nacional de General San Martín, Argentina) at 1:500 or 1:1000 (a condition that did not promote parasite agglutination or affected parasite motility) or an IgG negative control (rabbit pre-immune serum) at 1:1000. After each treatment, parasites were washed three times with RSB (1500 × ***g*** for 5 min at 4 °C) prior to the following experiments.

### Insects

*Rhodnius prolixus* were reared and maintained as previously described (Azambuja and Garcia, 1997) by the insectary of the Laboratório de Bioquímica e Fisiologia de Insetos, Instituto Oswaldo Cruz, FIOCRUZ. Briefly, fifth-instars larvae were randomly chosen, starved for 30 days after the last ecdysis and then allowed to feed on defibrinated rabbit blood through a membrane feeder. Ten days after feeding, the insects were dissected, the posterior midguts (small intestine) removed, longitudinally sectioned and washed three times in RSB to expose their luminal surfaces, as previously described (Gonzalez *et al*., 2011). Then, they were processed as described below.

### Ex vivo *interaction between* R. prolixus *dissected midgut and* T. cruzi

Tissue fragments from individual intestines were placed into 1.5 mL microtubes containing the treated parasites as described above, and then, incubated for 15 min at room temperature, under gentle shaking. Four dissected midguts were assayed per treatment. Afterwards, the explanted midguts were spread onto glass slides and the numbers of attached parasites per 100 randomly chosen epithelial cells in 10 different fields of each midgut explanted were quantified by counting under the light microscope (Gonzalez *et al*., 2011). Results are shown as the mean ± standard error of the mean of two experiments.

### In vivo *colonization of* R. prolixus *by* T. cruzi *and parasite re-isolation*

After a starvation period of 30 days, fifth-instars larvae were fed through a membrane feeder on defibrinated rabbit blood containing 9 × 10^6^ epimastigotes mL^−1^. In the 30th and 32nd days from feeding, the insects were washed and incubated in White's solution for 90 min (Bronfen *et al*., 1989) and subsequently washed abundantly in RSB. Afterwards, the rectal ampule was explanted through abdominal compression and homogenized in RSB. Feces content was analysed by optical microscopy. Then, the gut content from 10–15 insects were incubated in 25 cm^3^ flask culture containing BHI medium supplemented with streptomycin (100 *µ*g mL^−1^), penicillin (10 *µ*g mL^−1^), ampicillin (100 *µ*g mL^−1^), kanamycin (20 *µ*g mL^−1^) and gentamicin (140 *µ*g mL^−1^). The culture was checked daily for parasite, fungi and bacteria growth, and when required the following combined approaches were performed to eliminate contamination: centrifugation at low or high ***g*** force followed by inoculation in W tubes.

### Flow cytometry analysis

*Trypanosoma cruzi* epimastigotes (Dm28c) (3 × 10^6^ cells) were incubated or not with 0.5 *µ*m phenanthroline for 1 h or 24 h at 28 °C. Then, cells were washed three times in PBS and the cellular viability was confirmed by motility and lack of staining after challenging with Trypan blue. Treated cells were further monitored by assessing its growth in phenanthroline-free medium, after cellular normalization, the growth was similar to non-treated parasites (data not shown). Thereafter, cells were fixed for 30 min in 1% paraformaldehyde in PBS at room temperature, followed by extensive washing. Then, cells were incubated for 1 h at room temperature with anti-Tcgp63-I antibody (Cuevas *et al*., 2003) at 1:500 dilution, respectively. Cells were then incubated for an additional hour with 1:500 dilution of ALEXA 488- labelled goat anti-rabbit immunoglobulin G. Finally, cells were washed three times in PBS and analysed in a flow cytometry EPICS ELITE (Coulter Electronics, Hialeah, FL, USA) equipped with 15 mW argon laser emitting at 488 nm. In addition, the exposition of gp63 homologues in several *T. cruzi* isolates (G, Dm28c, Y and CL Brener) was compared by the analysis of the mean fluorescence intensity after anti-Tcgp63-I antibody incubation. Non-treated cells, those treated with the pre-immune serum and those treated with the secondary antibody alone were run in parallel as controls. Each experimental population was then mapped by using a two-parameter histogram of forward-angle light scatter *vs* side scatter. The mapped population (*n* = 10 000) was then analysed for log green fluorescence by using a single-parameter histogram.

### Western blotting analysis

Epimastigotes (1 × 10^9^ parasites) from Dm28c were collected by centrifugation at 3000 × ***g*** for 10 min at 4 °C, washed three times with cold PBS and lysed by six freezing and thawing cycles in liquid nitrogen at room temperature in a Tris-HCl 40 mm (pH 6.8) buffer containing 4% CHAPS, 0.5 mm TLCK, 1 mm PMSF and 100 *µ*m E-64, which were included to inhibit cruzipain and other cysteine peptidases and allow the analysis of minority peptidases. Then, cellular debris was removed by centrifugation at 16 000 × ***g*** for 10 min at 4 °C and the protein concentration of the supernatants was determined using BSA as standard (Lowry *et al*., 1951). This cellular extract was used for western blotting analysis, zymography and in solution proteolytic assays. For western blotting analysis, 10 *µ*g of the parasite extract was added to reducing SDS-PAGE sample buffer 1×, boiled for 5 min, and resolved by electrophoresis on a 10% SDS-PAGE under reducing conditions, as previously described (d'Avila-Levy *et al*., 2006*b*). Following electrophoresis, the membrane was blocked overnight in 10% skim milk dissolved in TBST. Subsequently, the membrane was incubated for 2 h with the rabbit anti-Tcgp63-I at 1:100 dilution. The secondary antibody used was peroxidase-conjugated goat anti-rabbit IgG at 1:25 000 (Pierce) followed by chemiluminescence immunodetection after reaction with ECL reagents.

### Zymography analysis

To this set of experiments, soluble extracts (10 *µ*g) from Dm28c were separated by 12% SDS-PAGE co-polymerized with 0.1% gelatin (Heussen and Dowdle, 1980). Samples were diluted (v/v) in SDS-PAGE sample buffer 4× without reducing agent and loaded onto gels. Electrophoresis was carried out under constant current (25 mA) at 4 °C. The gels were then soaked in 2.5% Triton X-100 under agitation for 1 h at room temperature for SDS removal and enzyme renaturation. The effect of pH on the proteolytic activity was determined by incubating the gels for 24 h at 37 °C in 50 mm phosphate buffer (pH 5.5 and 7.4) and 100 mm glycine-NaOH buffer pH 10.0 containing or not 2 mm DTT. Then, the gels were stained with 0.2% Coomassie Brilliant Blue R-250 in methanol:acetic acid:water (40:10:50) and destained in the same solution without the dye. The gels were scanned with the Image Scanner III (GE HealthCare) and analysed by the Image Master 2D Elite software (GE HealthCare). To determine the peptidase class, the lysates were pre-incubated for 30 min with the following inhibitors for 30 min at room temperature: pepstatin A (1 *µ*m) for aspartic-peptidases, phenanthroline (10 mm) for metallo-peptidases, PMSF (1 mm) for serine-peptidases and E-64 (10 *µ*m) for cysteine-peptidases. These inhibitors were also included in the pH 10.0 buffer in which the zymograms were incubated overnight.

### Enzyme assay in solution

Peptidase activity was measured continuously using the fluorogenic peptide substrate MCA-Pro-Cha-Gly-Nva-His-Ala-Dpa-NH2 at 37 °C in 0.1 M glycine-NaOH buffer containing 1 mm CaCl_2_ pH 10.0. The assays were performed in black round bottom 96-microwell plates in the Spectra Max Gemini spectrofluorimeter (Molecular Devices), with excitation wavelength at 328 nm and emission at 393 nm. The reaction started by the addition of 10 *µ*m substrate to the parasite extract (10 *µ*g) in a total volume of 100 *µ*L. The inhibition studies were performed by pre-incubation for 30 min at room temperature with the following inhibitors: 1 *µ*m pepstatin A, 10 mm phenanthroline, 1 mm PMSF or 10 *µ*m E-64. The assays were controlled for self-liberation of the fluorophore over the same time interval. All results were performed three times in triplicate, and are expressed as mean ± standard deviation.

### Statistical analysis

The data were analysed statistically using Student's *t* test or ANOVA using Prisma 6 GraphPad software. *P* values of 0.05 or less were considered statistically significant.

## Results

### Effects of divalent metal chelators on parasite viability

**Fig. 1.**
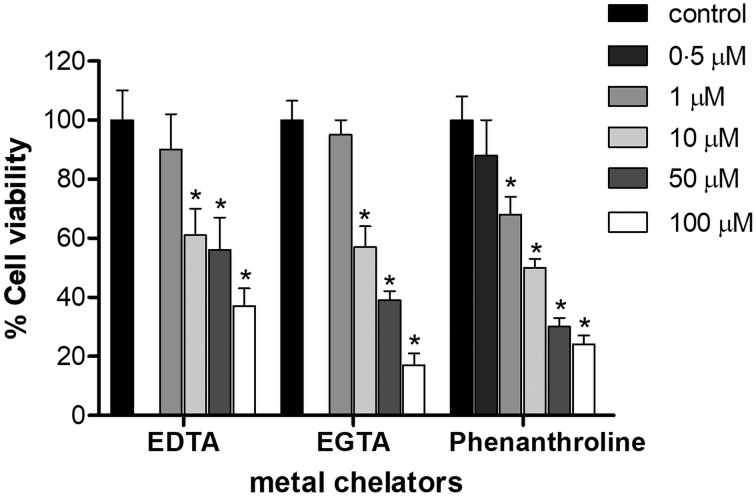
Effect of metal chelators on the viability of *T. cruzi* Dm28c epimastigotes. Parasites (2 × 10^7^ cells) were cultured for 1 h in the presence or absence of different concentration of either EDTA, EGTA or phenanthroline (0–100 *µ*m). The results correspond to the mean ± s.e.m. of three independent experiments performed in triplicate. An asterisk denotes values significantly different from control (untreated cells) using Student's *t* test (*P < 0.05).

*Trypanosoma cruzi* epimastigotes were incubated in the presence of EDTA, EGTA and phenanthroline for 1 h (Fig. 1). Concentration of 1 *µ*m for EDTA and EGTA, and 0.5 *µ*m for phenanthroline did not affect parasite viability compared to DMSO-treated controls under the assayed conditions (*P* > 0.05). The IC_50_ of EDTA, EGTA and phenanthroline was 7, 8 and 10 *µ*m, respectively (Fig. 1).

### Effect of divalent metal chelators and anti-Tcgp63-I antibodies on *T. cruzi*–*R. prolixus* interaction

**Fig. 2.**
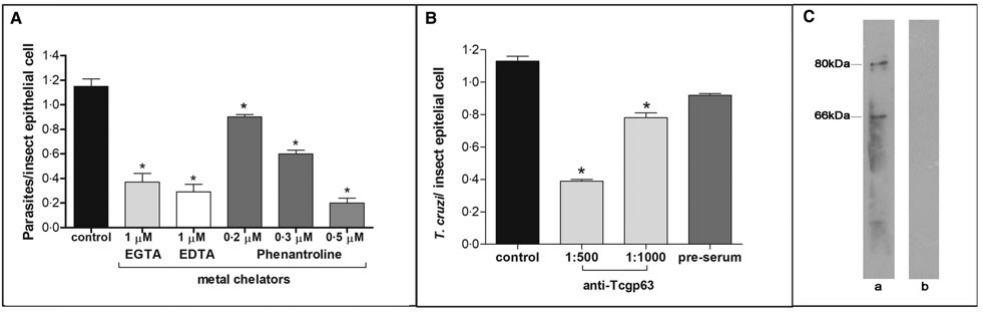
Effect of divalent metal chelators and anti-Tcgp63-I antibodies on *T. cruzi*–*R. prolixus* interaction. Dm28c epimastigotes (2 × 10^7^ cells) were treated for 1 h at 27 °C with 1 *µ*m EGTA, EDTA or phenanthroline at 0.2, 0.3 or 0.5 *µ*m (**A**). Alternatively, the parasites were treated for 60 min at room temperature with anti-Tcgp63-I antibodies at 1:500 and 1:1000 dilution, or pre-immune serum at 1:500 (**B**). Parasite viability was not affected by the treatments used in this set of experiments. Following interaction for 15 min with the insect gut, the number of adhered parasites/insect gut epithelial cells was estimated by randomly counting at least 100 epithelial cells in quadruplicate in 10 random fields. The results are shown as the mean ± s.e.m. of two independent experiments. An asterisk denotes significantly different from control (untreated cells) using Student's *t* test (*P < 0.001). Lastly, western blotting analyses identified two reactive bands against *T. cruzi* Dm28c epimastigotes (a), while no reactive molecules were recognized by the pre-immune serum (b) (**C**).

In this set of experiments, each divalent metal chelator was incubated with *T. cruzi* epimastigotes followed by their exposure to dissected posterior midgut of the insect. The metal chelators strongly impaired the parasite-gut binding by approximately 68, 75 and 83% for EDTA (1 *µ*m), EGTA (1 *µ*m) and phenanthroline (0.5 *µ*m), respectively, in comparison to control as shown in Fig. 2A. A distinguished feature of phenanthroline is that it chelates Zn^2+^ more avidly than EDTA and EGTA. This ion is crucial for maintaining leishmanial gp63 active site conformation and cannot be efficiently replaced by other divalent ions (Bouvier *et al*., 1989). Considering the structural similarities between *T. cruzi* and leishmanial gp63 molecules (El-Sayed and Donelson, 1997; Kulkarni *et al*., 2009; Abad-Franch *et al*., 2013), we evaluated the dose-dependent effect of phenanthroline on the proportion of parasite adhesion to the midgut. In phenanthroline doses ranging from 0.2 to 0.5 *µ*m, the adhesion of epimastigotes was reduced in the range of 39–83% in relation to the control (Fig. 2A), which supports the hypothesis that metallo-dependent enzymes play a role in the adhesion to *R. prolixus*. Supporting this hypothesis, the pre-treatment of parasites with anti-Tcgp63-I antibodies considerably reduced the interaction process, in relation to the control. The inhibition was 31 and 65% as antibody concentration rose from 1:1000 to 1:500, respectively (Fig. 2B). The antibody concentrations used did not promote parasite agglutination (data not shown). Parasites treated with the pre-immune serum adhered to the midguts at a rate similar to that of the control (Fig. 2B). Considering that the antibody was raised against *T. cruzi* CL Brener strain, we performed Western blotting against Dm28c epimastigote extracts, which revealed two reactive polypeptides migrating at 80 and 66 kDa (Fig. 2C).

### Modulation of Tcgp63-I levels after different stimulus

**Fig. 3.**
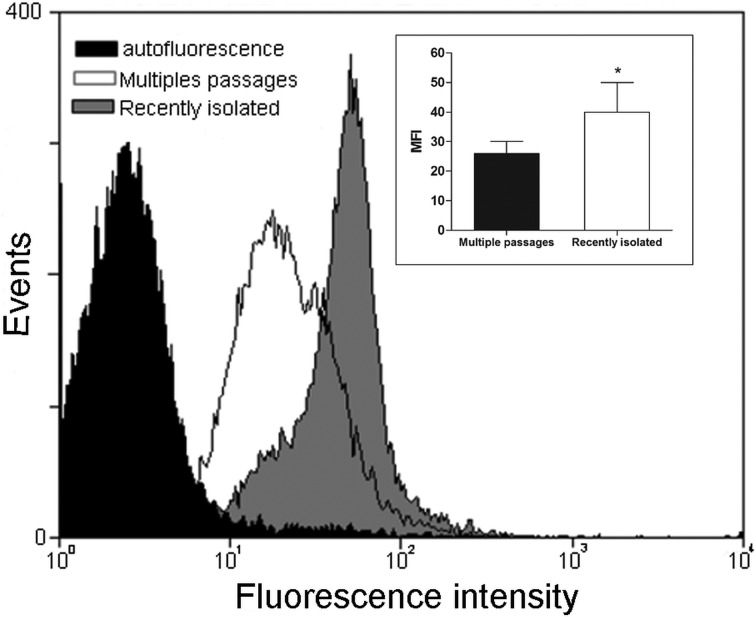
Tcgp63-I expression in *T. cruzi* Dm28c is enhanced after passage in *R. prolixus*. Cells were re-isolated in culture after insect colonization, as described in materials and methods. Long-term culture-adapted parasites (white) and recently isolated parasites (grey) were incubated in the presence of anti-Tcgp63-I antibody at 1:500 dilution and analysed by flow cytometry. Representative data of the analysis of 10 000 cells from one of three experiments are shown. The inset shows the mean of fluorescence intensity (MFI) levels. The asterisk indicates different values for MFI level between control and trypomastigotes re-isolated (*P < 0.05).

Aiming to determine the effect of insect passage on the expression levels of Tcgp63-I, we re-isolated parasites after 30 days from the initial infection. The rectal ampoules were analysed for parasite presence and either epimastigotes or trypomastigotes were visualized after insect abdominal compression (data not shown). Then, pure cultures were established as described in Materials and methods, and recently isolated from insect colonization or multiple passages parasites were incubated with anti-Tcgp63-I antibodies and analysed by flow cytometry (Fig. 3). Recently isolated parasites presented a significant increase in the mean of fluorescence intensity (MFI) levels of Tcgp63-I molecules in comparison to culture-adapted parasites (inset of Fig. 3).

Aiming to evaluate if phenanthroline would promote an imbalance in the exposition of Tcgp63-I from *T. cruzi*, parasites were treated with phenanthroline at 0.5 *µ*m for 1 h and assayed by flow cytometry using anti-Tcgp63-I. No statistical significance between treated and non-treated parasites was detected (data not shown). Therefore, we exposed the parasite for longer periods (up to 96 h) with phenanthroline at lower concentrations (0.25 *µ*m). Subsequently, we compared Tcgp63-I levels between treated parasites and control cells. Under the assayed conditions, no relevant change in FL-1 peak was detected (data not shown).

### Evaluation of Tcgp63-I levels in different *T. cruzi* strains

The adhesion rate to *R. prolixus* dissected midguts of different *T. cruzi* strains was previously determined by our research group (Uehara *et al*., 2012). To this end, we assayed Tcgp63-I levels in these strains, and no direct correlation was found between adhesion rate and anti-Tcgp63-I binding, since the G strain presented the strongest antibody recognition as assessed by MFI, and this strain presented the lowest binding rate among Dm28c, CL Brener and Y isolates (Uehara *et al*., 2012). In addition, no statistical difference in MFI was observed among these three isolates, while they clearly presented different binding rates to *R. prolixus* explanted midguts (Uehara *et al*., 2012) (Supplementary Material Fig. S1).

### Evaluation of enzyme activity

**Fig. 4.**
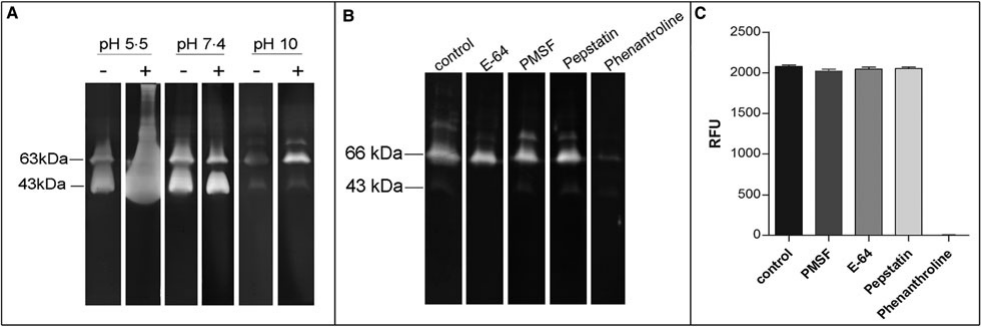
Effect of peptidase inhibitors, DTT and pH on the proteolytic activity of soluble extracts from *T. cruzi* Dm28c epimastigotes. Gelatin-SDS-PAGE were incubated in different buffers supplemented (+) or not (−) with DTT 2 mm at 37 °C for 24 h (**A**). To determine the enzymatic class, zymograms were incubated for 24 h in 100 mm glycine-NaOH buffer pH 10.0 in the absence (control) or presence of the following proteolytic inhibitors: 10 *µ*m E-64, 1 mm PMSF, 1 *µ*m pepstatin, or 10 mm phenanthroline (**B**). The numbers on the left indicate apparent molecular masses of the active bands expressed in kDa. To provide additional evidence on the enzyme class characterization, in solution assays were performed using the fluorogenic substrate MCA-Pro-Cha-Gly-Nva-His-Ala-Dpa-NH2 in 100 mm glycine-NaOH buffer pH 10.0, in the absence (control) or presence of the same inhibitors described above. Enzyme activity was expressed as micromoles of MCA per minute per micrograms of protein (**C**).

In order to confirm the presence of active metallopeptidases in *T. cruzi* extracts, we analysed gelatin degradation in zymography and cleavage of a fluorogenic peptide substrate by a parasite extract obtained under cysteine peptidase inhibition. Using zymography assay, we observed degradation haloes even after a strict protocol for cysteine peptidase inhibition in a wide pH range (5.5–10.0), with maximum activity observed at pH 5.5. The presence of DTT in the reaction buffer enhanced the proteolytic activity (Fig. 4A). Zymography analysis revealed two major bands migrating at approximately 43 and 63 kDa, which were strongly inhibited by phenanthroline (a reversible inhibitor of metal-dependent peptidases) (Fig. 4B). Densitometric analysis showed that the proteolytic activity is reduced by ~87% compared to control. PMSF (a reversible inhibitor of serine peptidases), E-64 (an irreversible inhibitor of cysteine peptidases) or pepstatin (a reversible inhibitor of aspartic peptidases) did not affect the proteolytic activity (data not shown). These results confirm the presence of active metallopeptidases in *T. cruzi* epimastigote extracts. We have also assayed the soluble extract with a fluorogenic substrate generally used for metallo-type peptidase characterization in the absence or presence of the same specific peptidase inhibitors described above. The substrate was readily hydrolysed by the soluble extract, and this proteolytic activity was completely inhibited only in the presence of phenanthroline, corroborating our previous findings (Fig. 4C).

## Discussion

Parasite peptidases are known to be involved in many cellular events, including interaction with both mammalian hosts and insect vectors (Garcia *et al*., 2010; Isnard *et al*., 2012; Santiago *et al*., 2017; Siqueira-Neto *et al*., 2018). In *T. cruzi*, metallopeptidases have been less studied and only in the last decade gp63 homologues, orthologues and pseudogenes were described (Kulkarni *et al*., 2009; Alvarez *et al*., 2012). To this end, the functional role of *T. cruzi* gp63 molecules is poor explored (d'Avila-Levy *et al*., 2014). The high heterogeneity of *T. cruzi GP63* genes creates a challenge to the study of this gene family. The first challenge in studying multigenic families is to classify the genes under study. Cuevas and colleagues (Cuevas *et al*., 2003) analysed the *T. cruzi* genome and identified several groups of genes that belong to the *GP63* family, with multiple members in each. In this sense, *GP63* genes were grouped in *Tcgp63-I* and *II*, which are present as high-copy-number genes, as well as *Tcgp63-III*, which are pseudogenes (Cuevas *et al*., 2003). At least four *GP63* mRNA, which belong to *Tcgp63-I*, are developmentally regulated in the different parasite stages, being more abundant in amastigotes than in epimastigotes or trypomastigotes (Grandgenett *et al*., 2000). Although *Tcgp63-II* genes are more abundant in the genome than *Tcgp63-I*, only the latter is detectable at the protein level and presents expressive mRNA levels in all developmental stages of CL Brener strain. Tcgp63-I is a proteolytically active enzyme bound to the membrane by a GPI anchor and with a possible role in the infection of host cells, since antibodies raised against a synthetic peptide derived from Tcgp63-I sequence partially blocked invasion of Vero cells by trypomastigotes (Cuevas *et al*., 2003).

Here, we demonstrated that protein homologues to Tcgp63-I are present in epimastigote extracts of *T. cruzi* clone Dm28. CL-Brener, to which the antibody was raised, belongs to discrete typing unit (DTU) VI, while the clone Dm28c to DTU I. The distinct *T. cruzi* DTUs present a previously underestimated genome complexity (Callejas-Hernandez *et al*., 2018) that is reflected in significant phenotypic variation and different behaviour both *in vitro* and *in vivo* in terms of pathophysiology, virulence, tropism and immunological responses (Rassi *et al*., 2012). In addition, we have also detected and partially characterized active metallopeptidases in this parasite strain, which are more active at acidic pH, are stimulated by DTT and inhibited by phenanthroline, characteristics shared by several metallopeptidases in trypanosomatids, including gp63 (d'Avila-Levy *et al*., 2003; Santos *et al*., 2006). A 65-kDa metallo-dependent enzyme has been previously detected in *T. cruzi* Dm28c, and parasite treatment with phenanthroline strongly inhibited metacyclogenesis (Bonaldo *et al*., 1991). Our data also demonstrated a clear impact on epimastigote proliferation caused by phenanthroline exposure, which is in accordance with previous report (Santos *et al*., 2012).

The exposure of parasites to the pressure of peptidase inhibitors influenced the protein content of the inhibited molecule or other abundant peptidases (Sangenito *et al*., 2009; Santos *et al*., 2009). Here, the zinc-chelator phenanthroline, even after 24 h exposure, did not promote any change in either cruzipain or Tcgp63-I levels. In addition, the possible role of Tcgp63-I or metallo-dependent enzymes on the interaction of *T. cruzi* with the midgut of triatomines has not been assessed up to now, although Tcgp63-I has a surface location and expression in epimastigotes, which is conceivable with a role in this part of the parasite life cycle (Cuevas *et al*., 2003). In this sense, the present study investigated the effect of metal chelators as well as the effect of the antibody raised by Cuevas *et al*. (2003) on the interaction of *T. cruzi* with *R. prolixus*, and both treatments considerably reduced the interaction of the parasite with the insect explanted guts.

The use of divalent metal chelators as an indirect approach to access gp63 functions in the insect interaction has been previously applied by our research group, for instance while assessing the interaction of *Leishmania infantum* and *L. braziliensis* with insect cell lines, as well as the live insect vectors, *Lutzomyia intermedia* and *Lu. longipalpis*, respectively (Soares *et al*., 2017), and the interaction of *Angomonas deanei*, *Crithidia fasciculata*, *Herpetomonas* spp., *Leptomonas* spp. with *Aedes aegypti* or *Aedes albopictus* and *Phytomonas* spp. with *Oncopeltus fasciatus* (d'Avila-Levy *et al*., 2006*a*, *b*; Nogueira de Melo *et al*., 2006; Pereira *et al*., 2009; Pereira *et al*., 2010). Either model insect hosts or the natural pairs were analysed (d'Avila-Levy *et al*., 2006*a*, *b*; Soares *et al*., 2017). Although phenanthroline can act on any metal-dependent cellular process, the bulk of evidence supports the role of metallopeptidases, particularly Tcgp63-I. Although it is still uncertain the mechanism of action of these molecules, a proteolytic independent role of gp63 has been demonstrated and an insect gut receptor was recognized by gp63 molecules in other trypanosomatids (d'Avila-Levy *et al*., 2006*b*).

To adapt and survive into extreme distinct environments like mammalian hosts and insects, a myriad of parasite proteins plays a concerted action. In *T. cruzi*, cruzipain, calpain-like molecules, GIPLs and GP72 seems to be involved in *T. cruzi* interaction with the invertebrate host (de Jesus *et al*., 1993; Basombrio *et al*., 2002; Nogueira *et al*., 2007; Ennes-Vidal *et al*., 2011). The molecular interactions that take place in the insect midgut that allows *T. cruzi* epimastigote binding and multiplication with further migration to the rectum and metacyclogenesis are certainly depend on multiple molecules. The study of isolated molecules helps but is insufficient to fully elucidate the functional impact of the complex structures that can be formed and are upon the influence of the microenvironment of the insect midgut. Interestingly, *T. cruzi* re-isolated after insect colonization presented an enhanced exposure of Tcgp63-I. *Roberts et al.* (1995) first described that passage of *L. chagasi* attenuated parasites through mice stimulated the expression of a surface 59-kDa glycoprotein (Roberts *et al*., 1995). Furthermore, *Sadlova et al.* (2006) showed experimentally that attenuated *L. major* parasites presented low enzymatic activity of gp63. However, these parasites presented a drastic enhancement in the proteolytic activity after passages through mice (Sadlova *et al*., 2006).

In conclusion, we provided for the first time evidence on the participation of Tcgp63-I and metallo-dependent enzymes on *T. cruzi* interaction with the insect vector, and demonstrated that the challenge that the parasite deals during insect vector colonization induces the enhanced production of Tcgp63-I. Although, the precise molecular mechanism underlying the roles of these molecules in the vector interaction still needs further research, it sheds some light on a neglected interface: the parasite–insect interaction.

## Supplementary material

For supplementary material accompanying this paper visit http://dx.doi.org/10.1017/S0031182019000441. click here to view supplementary material

## References

[ref1] Abad-Franch F, Diotaiuti L, Gurgel-Goncalves R and Gurtler RE (2013) Certifying the interruption of Chagas disease transmission by native vectors: cui bono? Memorias do Instituto Oswaldo Cruz 108, 251–254. 23579810 10.1590/0074-0276108022013022 PMC3970656

[ref2] Alvarez VE, Niemirowicz GT and Cazzulo JJ (2012) The peptidases of *Trypanosoma cruzi*: digestive enzymes, virulence factors, and mediators of autophagy and programmed cell death. Biochimica et Biophysica Acta 1824, 195–206. 21621652 10.1016/j.bbapap.2011.05.011

[ref19] Azambuja P and Garcia ES (1997) Care and maintenance of triatomine colonies In Crampton JM, Beard CB and Loius C (eds), Molecular Biology of Insect Disease Vectors: a methods manual. London: Chapman and Hall, pp. 56–64.

[ref3] Azambuja P, Ratcliffe NA and Garcia ES (2005) Towards an understanding of the interactions of *Trypanosoma cruzi* and *Trypanosoma rangeli* within the reduviid insect host *Rhodnius prolixus*. Anais da Academia Brasileira de Ciencias 77, 397–404. 16127548 10.1590/s0001-37652005000300004

[ref4] Basombrio MA, Gomez L, Padilla AM, Ciaccio M, Nozaki T and Cross GA (2002) Targeted deletion of the gp72 gene decreases the infectivity of *Trypanosoma cruzi* for mice and insect vectors. Journal of Parasitology 88, 489–493. 12099416 10.1645/0022-3395(2002)088[0489:TDOTGG]2.0.CO;2

[ref5] Bern C, Kjos S, Yabsley MJ and Montgomery SP (2011) *Trypanosoma cruzi* and Chagas’ disease in the United States. Clinical Microbiology Reviews 24, 655–681. 21976603 10.1128/CMR.00005-11 PMC3194829

[ref6] Bonaldo MC, d'Escoffier LN, Salles JM and Goldenberg S (1991) Characterization and expression of proteases during *Trypanosoma cruzi* metacyclogenesis. Experimental Parasitology 73, 44–51. 2055300 10.1016/0014-4894(91)90006-i

[ref7] Bouvier J, Bordier C, Vogel H, Reichelt R and Etges R (1989) Characterization of the promastigote surface protease of *Leishmania* as a membrane-bound zinc endopeptidase. Molecular and Biochemical Parasitology 37, 235–245. 2608099 10.1016/0166-6851(89)90155-2

[ref8] Branquinha MH, Marinho FA, Sangenito LS, Oliveira SS, Goncalves KC, Ennes-Vidal V, d'Avila-Levy CM and Santos ALS (2013) Calpains: potential targets for alternative chemotherapeutic intervention against human pathogenic trypanosomatids. Current Medicinal Chemistry 20, 3174–3185. 23899207 10.2174/0929867311320250010 PMC4181241

[ref9] Branquinha MH, Oliveira SS, Sangenito LS, Sodre CL, Kneipp LF, d'Avila-Levy CM and Santos ALS (2015) Cruzipain: an update on its potential as chemotherapy target against the human pathogen *Trypanosoma cruzi*. Current Medicinal Chemistry 22, 2225–2235. 25994861 10.2174/0929867322666150521091652

[ref10] Bronfen E, de Assis Rocha FS, Machado GB, Perillo MM, Romanha AJ and Chiari E (1989) Isolation of *Trypanosoma cruzi* samples by xenodiagnosis and hemoculture from patients with chronic Chagas’ disease. Memorias do Instituto Oswaldo Cruz 84, 237–240. 2517566 10.1590/s0074-02761989000200012

[ref11] Callejas-Hernandez F, Rastrojo A, Poveda C, Girones N and Fresno M (2018) Genomic assemblies of newly sequenced *Trypanosoma cruzi* strains reveal new genomic expansion and greater complexity. Scientific Reports 8, 14631. 30279473 10.1038/s41598-018-32877-2 PMC6168536

[ref12] Cazzulo JJ, Cazzulo Franke MC, Martinez J and Franke de Cazzulo BM (1990) Some kinetic properties of a cysteine proteinase (cruzipain) from *Trypanosoma cruzi*. Biochimica et Biophysica Acta 1037, 186–191. 2407295 10.1016/0167-4838(90)90166-d

[ref13] Chaudhuri G and Chang KP (1988) Acid protease activity of a major surface membrane glycoprotein (gp63) from *Leishmania mexicana* promastigotes. Molecular and Biochemical Parasitology 27, 43–52. 3278222 10.1016/0166-6851(88)90023-0

[ref14] Cuevas IC, Cazzulo JJ and Sanchez DO (2003) Gp63 homologues in *Trypanosoma cruzi*: surface antigens with metalloprotease activity and a possible role in host cell infection. Infection and Immunity 71, 5739–5749. 14500495 10.1128/IAI.71.10.5739-5749.2003 PMC201075

[ref15] d'Avila-Levy CM, Souza RF, Gomes RC, Vermelho AB and Branquinha MH (2003) A metalloproteinase extracellularly released by *Crithidia deanei*. Canadian Journal of Microbiology 49, 625–632. 14663496 10.1139/w03-081

[ref16] d'Avila-Levy CM, Dias FA, Nogueira de Melo A, Martins J, Lopes AHCS, Santos ALS, Vermelho AB and Branquinha MH (2006 *a*) Insights into the role of gp63-like proteins in lower trypanosomatids. FEMS Microbiology Letters 254, 149–156. 16451193 10.1111/j.1574-6968.2005.00022.x

[ref17] d'Avila-Levy CM, Altoe EC, Uehara LA, Branquinha MH and Santos ALS (2014) GP63 function in the interaction of trypanosomatids with the invertebrate host: facts and prospects. Subcellular Biochemistry 74, 253–270. 24264249 10.1007/978-94-007-7305-9_11

[ref18] d'Avila-Levy CM, Santos LO, Marinho FA, Dias FA, Lopes AH, Santos ALS and Branquinha MH (2006 *b*) Gp63-like molecules in *Phytomonas serpens*: possible role in the insect interaction. Current Microbiology 52, 439–444. 16732452 10.1007/s00284-005-0222-8

[ref20] de Jesus AR, Cooper R, Espinosa M, Gomes JE, Garcia ES, Paul S and Cross GA (1993) Gene deletion suggests a role for *Trypanosoma cruzi* surface glycoprotein GP72 in the insect and mammalian stages of the life cycle. Journal of Cell Science 106(Pt 4), 1023–1033. 8126090 10.1242/jcs.106.4.1023

[ref21] El-Sayed NM and Donelson JE (1997) African trypanosomes have differentially expressed genes encoding homologues of the *Leishmania* GP63 surface protease. Journal of Biological Chemistry 272, 26742–26748. 9334260 10.1074/jbc.272.42.26742

[ref22] Ennes-Vidal V, Menna-Barreto RF, Santos ALS, Branquinha MH and d'Avila-Levy CM (2011) MDL28170, a calpain inhibitor, affects *Trypanosoma cruzi* metacyclogenesis, ultrastructure and attachment to *Rhodnius prolixus* midgut. PLoS One 6, e18371. 21483751 10.1371/journal.pone.0018371 PMC3070728

[ref23] Garcia ES, Genta FA, Azambuja P and Schaub GA (2010) Interactions between intestinal compounds of triatomines and *Trypanosoma cruzi*. Trends in Parasitology 26, 499–505. 20801082 10.1016/j.pt.2010.07.003

[ref24] Gonzalez MS, Silva LC, Albuquerque-Cunha JM, Nogueira NF, Mattos DP, Castro DP, Azambuja P and Garcia ES (2011) Involvement of sulfated glycosaminoglycans on the development and attachment of *Trypanosoma cruzi t*o the luminal midgut surface in the vector, *Rhodnius prolixus*. Parasitology 138, 1870–1877. 21902871 10.1017/S0031182011001521

[ref25] Grandgenett PM, Coughlin BC, Kirchhoff LV and Donelson JE (2000) Differential expression of GP63 genes in *Trypanosoma cruzi*. Molecular and Biochemical Parasitology 110, 409–415. 11071294 10.1016/s0166-6851(00)00275-9

[ref26] Heussen C and Dowdle EB (1980) Electrophoretic analysis of plasminogen activators in polyacrylamide gels containing sodium dodecyl sulfate and copolymerized substrates. Analytical Biochemistry 102, 196–202. 7188842 10.1016/0003-2697(80)90338-3

[ref27] Isnard A, Shio MT and Olivier M (2012) Impact of *Leishmania* metalloprotease GP63 on macrophage signaling. Frontiers in Cellular and Infection Microbiology 2, 72. 22919663 10.3389/fcimb.2012.00072 PMC3417651

[ref28] Kulkarni MM, Olson CL, Engman DM and McGwire BS (2009) *Trypanosoma cruzi* GP63 proteins undergo stage-specific differential posttranslational modification and are important for host cell infection. Infection and Immunity 77, 2193–2200. 19273559 10.1128/IAI.01542-08 PMC2681764

[ref29] Lowry OH, Rosebrough NJ, Farr AL and Randall RJ (1951) Protein measurement with the folin phenol reagent. Journal of Biological Chemistry 193, 265–275. 14907713

[ref30] Nogueira de Melo AC, d'Avila-Levy CM, Dias FA, Armada JL, Silva HD, Lopes AHCS, Santos ALS, Branquinha MH and Vermelho AB (2006) Peptidases and gp63-like proteins in *Herpetomonas megaseliae*: possible involvement in the adhesion to the invertebrate host. International Journal for Parasitology 36, 415–422. 16500661 10.1016/j.ijpara.2005.12.006

[ref31] Nogueira NF, Gonzalez MS, Gomes JE, de Souza W, Garcia ES, Azambuja P, Nohara LL, Almeida IC, Zingales B and Colli W (2007) *Trypanosoma cruzi:* involvement of glycoinositolphospholipids in the attachment to the luminal midgut surface of *Rhodnius prolixus*. Experimental Parasitology 116, 120–128. 17306256 10.1016/j.exppara.2006.12.014

[ref32] Noireau F, Diosque P and Jansen AM (2009) *Trypanosoma cruzi*: adaptation to its vectors and its hosts. Veterinary Research 40, 26. 19250627 10.1051/vetres/2009009 PMC2695024

[ref33] Olivier M, Atayde VD, Isnard A, Hassani K and Shio MT (2012) Leishmania virulence factors: focus on the metalloprotease GP63. Microbes and Infection 14, 1377–1389. 22683718 10.1016/j.micinf.2012.05.014

[ref34] Pereira FM, Bernardo PS, Dias Junior PF, Silva BA, Romanos MT, d'Avila-Levy CM, Branquinha MH and Santos ALS (2009) Differential influence of gp63-like molecules in three distinct *Leptomonas* species on the adhesion to insect cells. Parasitology Research 104, 347–353. 18830631 10.1007/s00436-008-1202-2

[ref35] Pereira FM, Dias FA, Elias CG, d'Avila-Levy CM, Silva CS, Santos-Mallet JR, Branquinha MH and Santos ALS (2010) Leishmanolysin-like molecules in *Herpetomonas samuelpessoai* mediate hydrolysis of protein substrates and interaction with insect. Protist 161, 589–602. 20359946 10.1016/j.protis.2010.02.001

[ref36] Perez-Molina JA and Molina I (2018) Chagas disease. Lancet 391, 82–94. 28673423 10.1016/S0140-6736(17)31612-4

[ref37] Rassi A Jr, Rassi A and Marcondes de Rezende J (2012) American trypanosomiasis (Chagas disease). Infectious Disease Clinics of North America 26, 275–291. 22632639 10.1016/j.idc.2012.03.002

[ref38] Roberts SC, Wilson ME and Donelson JE (1995) Developmentally regulated expression of a novel 59-kDa product of the major surface protease (Msp or gp63) gene family of *Leishmania chagasi*. Journal of Biological Chemistry 270, 8884–8892. 7721796 10.1074/jbc.270.15.8884

[ref39] Sadlova J, Volf P, Victoir K, Dujardin JC and Votypka J (2006) Virulent and attenuated lines of *Leishmania major*: DNA karyotypes and differences in metalloproteinase GP63. Folia Parasitol (Praha) 53, 81–90. 16898121

[ref40] Salazar R, Castillo-Neyra R, Tustin AW, Borrini-Mayori K, Naquira C and Levy MZ (2015) Bed bugs (*Cimex lectularius*) as vectors of *Trypanosoma cruzi*. American Journal of Tropical Medicine and Hygiene 92, 331–335. 25404068 10.4269/ajtmh.14-0483 PMC4347337

[ref41] Sangenito LS, Ennes-Vidal V, Marinho FA, Da Mota FF, Santos ALS, d'Avila-Levy CM and Branquinha MH (2009) Arrested growth of *Trypanosoma cruzi* by the calpain inhibitor MDL28170 and detection of calpain homologues in epimastigote forms. Parasitology 136, 433–441. 19250597 10.1017/S0031182009005629

[ref42] Santiago PB, de Araujo CN, Motta FN, Praca YR, Charneau S, Bastos IM and Santana JM (2017) Proteases of haematophagous arthropod vectors are involved in blood-feeding, yolk formation and immunity – a review. Parasites & Vectors 10, 79. 28193252 10.1186/s13071-017-2005-z PMC5307778

[ref43] Santos ALS, Branquinha MH and d'Avila-Levy CM (2006) The ubiquitous gp63-like metalloprotease from lower trypanosomatids: in the search for a function. Anais da Academia Brasileira de Ciencias 78, 687–714. 17143406 10.1590/s0001-37652006000400006

[ref44] Santos LO, Marinho FA, Altoe EF, Vitorio BS, Alves CR, Britto C, Motta MC, Branquinha MH, Santos ALS and d'Avila-Levy CM (2009) HIV aspartyl peptidase inhibitors interfere with cellular proliferation, ultrastructure and macrophage infection of *Leishmania amazonensis*. PLoS One 4, e4918. 19325703 10.1371/journal.pone.0004918 PMC2656615

[ref45] Santos ALS, Sodre CL, Valle RS, Silva BA, Abi-Chacra EA, Silva LV, Souza-Goncalves AL, Sangenito LS, Goncalves DS, Souza LO, Palmeira VF, d'Avila-Levy CM, Kneipp LF, Kellett A, McCann M and Branquinha MH (2012) Antimicrobial action of chelating agents: repercussions on the microorganism development, virulence and pathogenesis. Current Medicinal Chemistry 19, 2715–2737. 22455582 10.2174/092986712800609788

[ref46] Siqueira-Neto JL, Debnath A, McCall LI, Bernatchez JA, Ndao M, Reed SL and Rosenthal PJ (2018) Cysteine proteases in protozoan parasites. PLoS Neglected Tropical Diseases 12, e0006512. 30138453 10.1371/journal.pntd.0006512 PMC6107107

[ref47] Soares RP, Altoe ECF, Ennes-Vidal V, da Costa SM, Rangel EF, de Souza NA, da Silva VC, Volf P and d'Avila-Levy CM (2017) In vitro inhibition of *Leishmania* attachment to Sandfly Midguts and LL-5 Cells by Divalent Metal Chelators, Anti-gp63 and Phosphoglycans. Protist 168, 326–334. 28472733 10.1016/j.protis.2017.03.004

[ref48] Uehara LA, Moreira OC, Oliveira AC, Azambuja P, Lima AP, Britto C, Santos ALS, Branquinha MH and d'Avila-Levy CM (2012) Cruzipain promotes *Trypanosoma cruzi* adhesion to *Rhodnius prolixus* midgut. PLoS Neglected Tropical Diseases 6, e1958. 23272264 10.1371/journal.pntd.0001958 PMC3521651

[ref49] Waleckx E, Gourbiere S and Dumonteil E (2015) Intrusive versus domiciliated triatomines and the challenge of adapting vector control practices against Chagas disease. Memorias do Instituto Oswaldo Cruz 110, 324–338. 25993504 10.1590/0074-02760140409 PMC4489470

