# Supplementary figures and images for: Participation of Trypanosoma cruzi gp63 molecules on the interaction with Rhodnius prolixus
Source: Parasitology. 2019 May 6;146(8):1075–82. doi: 10.1017/S0031182019000441 (PMC6604109; doi:10.1017/S0031182019000441)

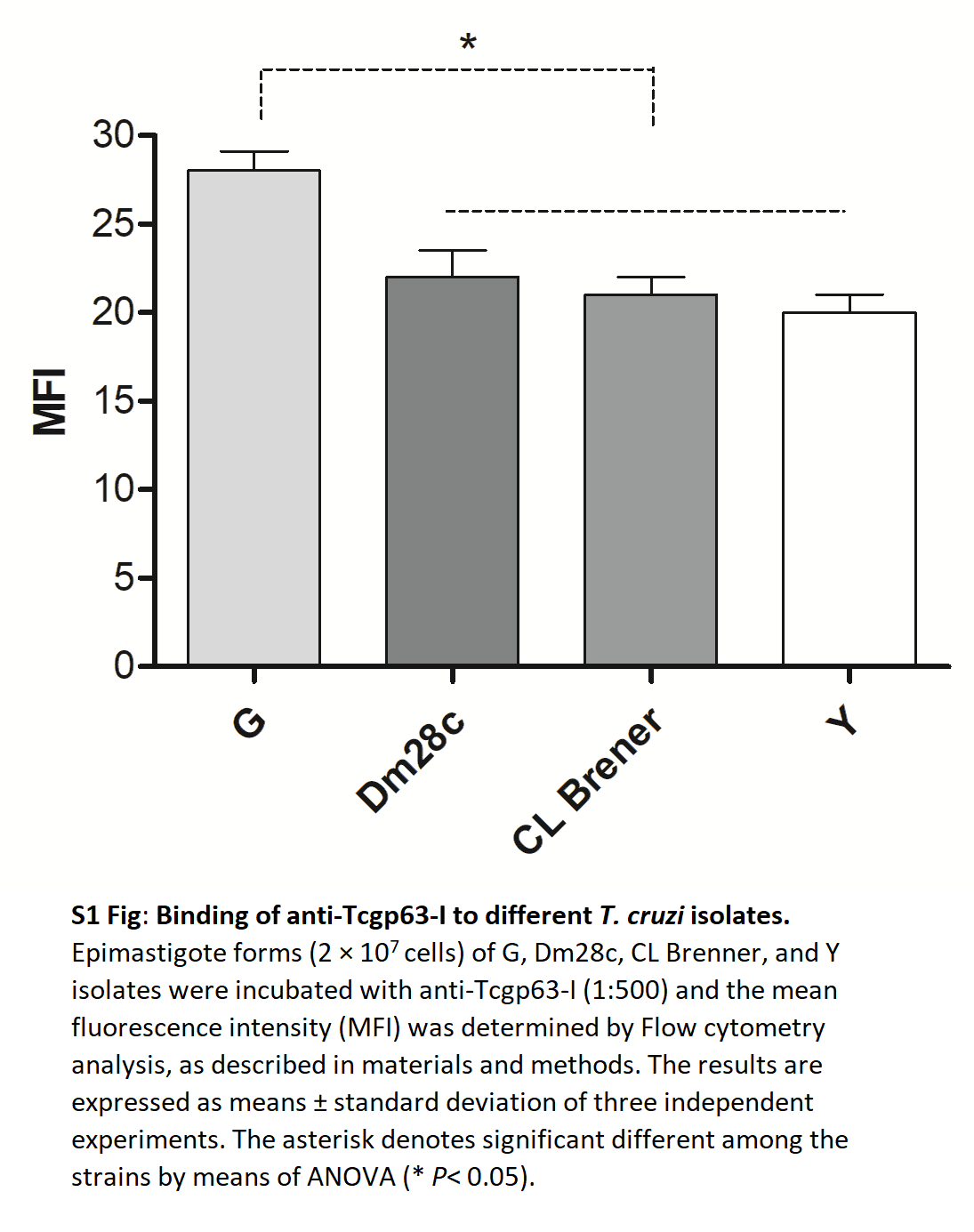

Supplement: Supplementary file 1 [file S0031182019000441sup001.tif]
